# Research progress of coumarins and their derivatives in the treatment of diabetes

**DOI:** 10.1080/14756366.2021.2024526

**Published:** 2022-01-24

**Authors:** Yinbo Pan, Teng Liu, Xiaojing Wang, Jie Sun

**Affiliations:** a School of Pharmacy and Pharmaceutical Sciences, Shandong First Medical University, Jinan, Shandong, China; b Institute of Materia Medica, Shandong Academy of Medical Sciences, Jinan, Shandong, China

**Keywords:** Diabetes, coumarin, target spots, biological activity

## Abstract

Diabetes is a group of metabolic diseases characterised by chronic hyperglycaemia caused by multiple causes, which is caused by insulin secretion and/or utilisation defects. It is characterised by increased fasting and postprandial blood glucose levels due to insulin deficiency or insulin resistance. It is reported that the harm of diabetes mainly comes from its complications, and the cardiovascular disease caused by diabetes is the primary cause of its harm. China has the largest number of diabetic patients in the world, and the prevention and control of diabetes are facing great challenges. In recent years, many kinds of literature have been published abroad, which have proved that coumarin and its derivatives are effective in the treatment of diabetic complications such as nephropathy and cardiovascular disease. In this paper, the types of antidiabetic drugs and the anti-diabetic mechanism of coumarins were reviewed.

## Introduction

Diabetes mellitus (DM) is a chronic metabolic disease characterised by the imbalance of glucose homeostasis, which leads to the increase of glucose levels in the blood. In recent decades, the incidence of diabetes has risen sharply all over the world, and the study of small molecules with potent antidiabetic activity is one of the most interesting research fields^1–3^.

**Figure 1.**
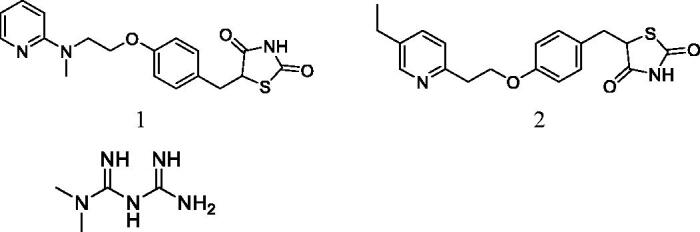
Various insulin sensitisers represent drugs (Compound **1** Rosiglitazone, Compound **2** Pioglitazone, Compound **3** Metformin).

At present, the hypoglycaemic agents in clinical use include incretin^4–10^ and insulin sensitizers^11–14^. In addition, there are other types of substances used to lower blood glucose, such as AMP-activated protein kinase AMPK^15^, α-glucosidase inhibitors^16^, amylases, and insulin analogues^17^. However, among the many marketed drugs, there are more or less some side effects while lowering blood glucose (Figure 1). Therefore, there is an urgent need to discover new drugs to compensate for or replace the shortcomings of current drugs. In recent years, more and more attention has been paid to the study of natural products. Coumarin compounds stand out in the process of drug research and development because of their advantages of multiple targets and less toxic side effects.

**Figure 2.**
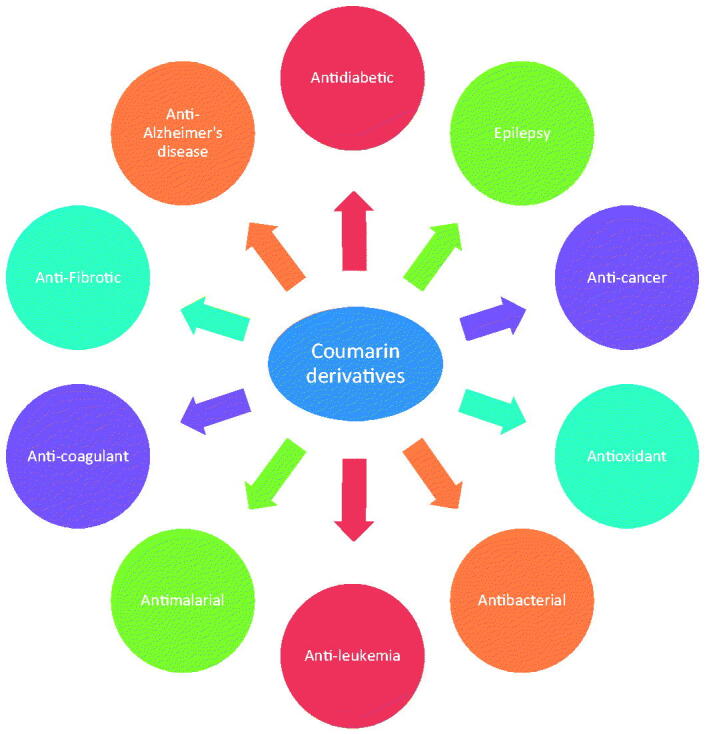
Biological activity of coumarin and its derivatives.

**Figure 3.**
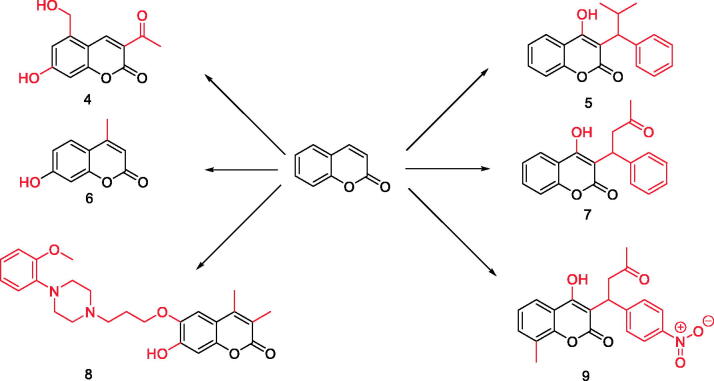
Various biological activities of coumarin and its derivatives (Compound **4–9**).

**Figure 4.**
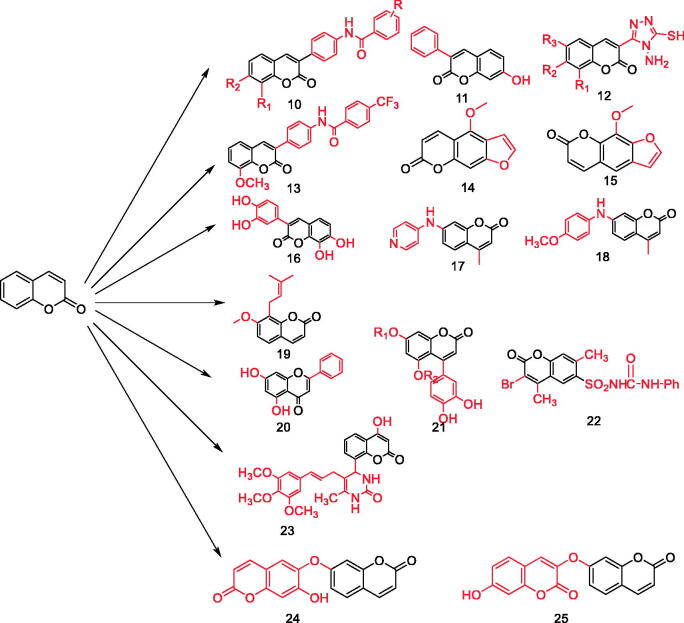
Various coumarins with hypoglycaemic function (Compound **10–25**).

The development or discovery of new highly effective drugs with few toxic side effects is the main goal of modern medicinal chemists. In recent years, coumarin compounds have received increasing attention. Coumarin and its derivatives have extensive biological activities, anticoagulant^18–21^, antibacterial^22–24^, anti-inflammatory^25–28^, antioxidant^29–31^, antitumor^32–35^, antiviral^36–39^, and enzyme inhibition effect^40–45^ (Figure 2). Coumarins are the general name of cis-o-hydroxy cinnamic acid lactones, which all have the basic skeleton of the benzo α-pyranone mother nucleus. Among the various toxic activities found on it, it has a less harmful effect on normal cells, especially in anti-diabetes, and has become one of the hotspots of drug research in the future (Figure 3). Recent studies have found that coumarins have significant effects in inhibiting α-glucosidase, AGE-RAGE signalling pathway, activating PPARγ and anti-oxidation, etc. (Table 1, Figure 4). In the following part of this review, I will present the hypoglycaemic effects of coumarins on different targets discovered to date.

**Table 1.** Coumarins and their different structures.

Target spot	Function	Coumarin species
α-glucosidase	Hydrolyzing glycosidic bonds in various sugar-containing compounds, it can degrade polysaccharides such as starch, maltose and sucrose into monosaccharides.	3-(4′-benzoyl amino-phenyl) coumarin derivatives; 3-aryl coumarin; 1,2,4-triazoles; Coumarin-1,2,3-triazole-acetamide hybrid derivative; Coumarin-cinnamic acid conjugate
AGE-RAGE	AGE is one of the key factors to induce diabetes and its complications, AGEs can accelerate the ageing of the human body and can cause various chronic degenerative diseases.	3-(4′-benzoyl amino-phenyl) coumarin derivatives; 3-aryl coumarin; Furan coumarin
Oxidative stress	In vitro antioxidant evaluation of antioxidant components; ROS is produced by hyperglycaemia, which causes damage to macromolecules and produces signal molecules.	4-methyl-7- aminocoumarin; 3-aryl coumarin; Pyrazole cyclic coumarin; Daidzein; Dicoumarins
Up-regulated expression of P2X3 after treatment	Non-selective ligand-gated cation channel, P2X3 receptors are involved in many neuropathic pain processes including DNP.	Osthole, etc
Activate PPAR-γ	Key regulatory factors of glucose metabolism, it can inhibit the AGE-RAGE system by activating PPAR-γ activity, thereby regulating oxidative stress.	Coumarin analogues such as Chrysin and Luteolin; 4-arylcoumarin glycoside; Coumarin-sulfonylurea conjugate
Insulin receptor	It is a tyrosine kinase transmembrane receptor that is effectively involved in the regulation of glucose homeostasis through insulin-bound phosphorylation.	Coumarin chalcones

### Source

Over the years, great attention has been paid to coumarin and its derivatives, which are versatile molecules exhibiting a wide variety of biological properties including antimicrobial, antiviral, anticancer, antioxidant, anti-inflammatory, anti-tuberculosis, anti-influenza, anti-Alzheimer, and anti-hyperlipidemia activities^46^. Coumarins are the general name of cis-o-hydroxy cinnamic acid lactones, which all have the basic skeleton of the benzo α-pyranone mother nucleus^47^. Coumarins are widely distributed in roots, stems, leaves, flowers, fruits and seeds of higher plants, especially in Umbelliferae, Rutaceae, Daphne and Oleaceae, and a few of them are found in microorganisms and animals. Some coumarin compounds can also be used for artificial synthesis.

### Active targets

The pathogenesis of diabetes is complex, and coumarin acts on different diabetes-related targets in multiple ways, thereby exerting a role in treating or improving diabetes-related symptoms.

#### α-Glucosidase

**Figure 5.**
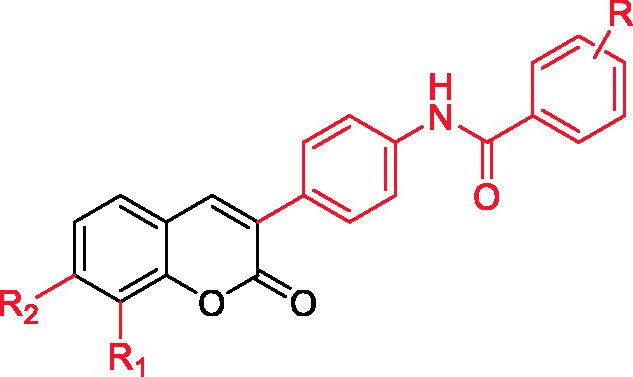
3-(4'-benzoyl amino-phenyl) coumarin derivatives.

α-glucosidase is widely distributed in the brush border of the small intestinal mucosa, which has an important influence on the structure of glycosyl groups. They can degrade polysaccharides such as starch, maltose and sucrose into monosaccharides^48^. It hydrolyses the glycosidic bonds in various sugar compounds by endo or exo cleavage, causing the increase of blood glucose. After hydrolysis, the sugary compounds mainly exist in the following three forms: monosaccharide, oligosaccharide and carbohydrate complex. The existing traditional α-glucosidase inhibitors, such as acarbose and voglibose, have more or less gastrointestinal adverse reactions such as nausea and vomiting. Hu et al.^49^^,^^50^ studied 3–(4′-benzoyl amino-phenyl) coumarin derivatives (Figure 5, Table 2), and found that their inhibitory activities on α-glucosidase were different from those of positive control drugs, but their inhibitory activities were all lower than 65 μmol/L, and compound **27** had stronger inhibitory activities through screening.

**Table 2.** 3-(4'-benzoylamino-phenyl) coumarin derivatives (Compound **26–35**).

Compound	*R*_1_	*R*_2_	*R*
**26**	H	OH	3-CH_3_
**27**	H	OH	3-Cl
**28**	H	OH	2-F
**29**	H	OH	4-Cl
**30**	H	OH	2-CH_3_,3-CH_3_
**31**	0CH_3_	H	4-F
**32**	OCH_3_	H	4-CF_3_
**33**	OCH_3_	H	3-Cl
**34**	OCH_3_	H	2-Cl,3-Cl
**35**	OH	H	4-CH_2_Cl

**Figure 6.**
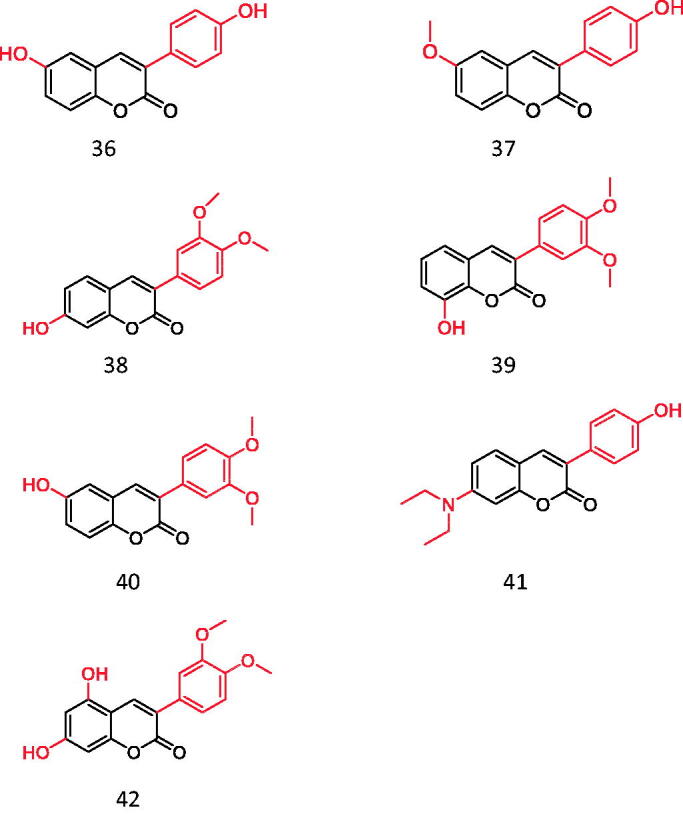
3-arylcoumarin (Compound **36–42**).

Wang et al. screened more than 40 kinds of 3-aryl coumarins (Figure 6). By comparing IC_50_ values, it was found that 3-aryl coumarins containing hydroxyl at position 7 showed strong α-glucosidase inhibitory activity, and 5,7-dihydroxy was more active than 7-hydroxy. Substituting 4′-OH on the benzene ring was another active site for inhibiting α-glucosidase. The scoring function of the best binding mode between acarbose and α-glucosidase was −129.508, and the lower scoring function showed that the ligand and protein had a high matching degree and the complex had high stability. The scores of the following compounds are all lower than −100. These seven compounds are almost completely embedded in the grid, and their conformation fits well with the binding pocket, thus achieving the best structure matching.

**Figure 7.**
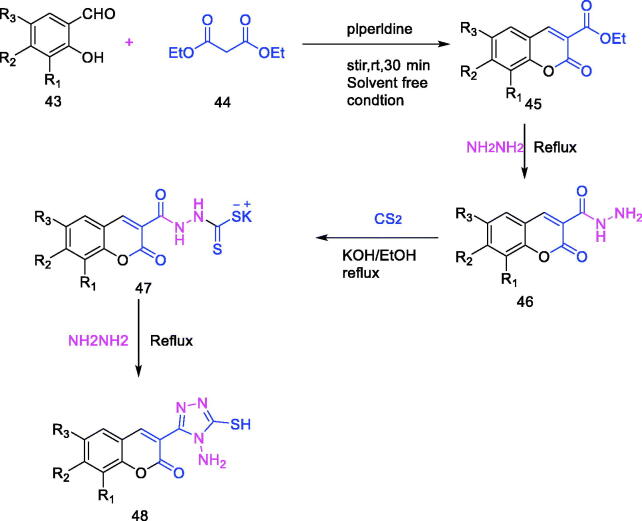
Synthesis of 1, 2, 4-Triazole coumarin hybrids.

Among heterocyclic compounds, coumarin and triazole have attracted more attention because of their natural existence and great biological activity. Five-membered heterocycles, especially 1,2,4-triazoles, play a key role in pharmaceutical chemistry due to their multiple uses. Vagish Channa Basappa et al.^1^ synthesised a series of coumarin-triazole hybrids (Figure 7, Table 3), and screened their inhibitory effects on α-amylase. The results showed that compounds **48–3** and **48–5** exhibited good inhibition effects on enzymes, and they were expected to become lead compounds for diabetes drugs.

**Table 3.** 1, 2, 4-Triazole coumarin hybrids.

Compound	*R*_1_	*R*_2_	*R*_3_
**48-1**	H	H	Br
**48-2**	H	H	Cl
**48-3**	OCH_3_	H	H
**48-4**	H	OCH_3_	H
**48-5**	OC_2_H_5_	H	H

**Table 4.** Coumarin-1,2,3-triazole-acetamide hybrid derivatives.

Compound	*N*	*R*
**53-1**	0	H
**53-2**	0	4-CH_3_
**53-3**	0	4-OCH_3_
**53-4**	0	4-F
**53-5**	0	4-2,4-diF
**53-6**	0	2-Cl
**53-7**	0	2,3-diCl
**53-8**	0	3-Br
**53-9**	0	4-Br
**53-10**	0	2-CH_3_ 3-Cl
**53-11**	0	2-CH_3_ 3-NO_2_
**53-12**	1	H
**53-13**	1	4-F

**Figure 8.**
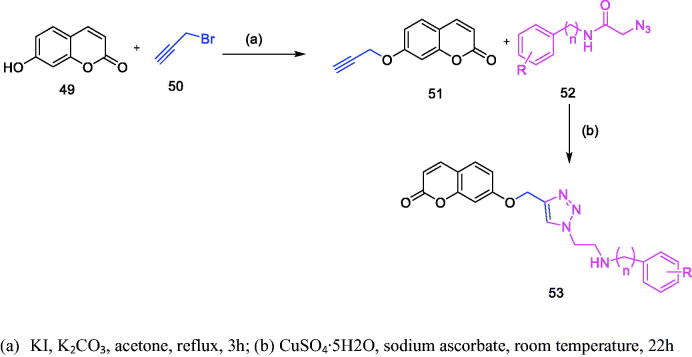
Synthesis of Coumarin-1,2,3-triazole-acetamide hybrid derivatives.

Nima Sepehri et al. synthesised coumarin-1,2,3-triazole-acetamide hybrid derivatives (Figure 8, Table 4) and evaluated their α-glucosidase inhibitory activity. It was found that all the coumarin-1,2,3-triazole-acetamide hybridised derivatives were superior to acarbose in inhibiting α-glucosidase, especially compound **h** and **d** were superior to other similar derivatives in inhibiting α-glucosidase^51^. Compound **h** interacts with the important residues Arg312, Asn241, Glu304, Ser308 and Pro309 of α-glucosidase active site. Compound **d** interacts with the important residues His201, Ile235 and Tyr151 in the active site of α-amylase.

**Figure 9.**
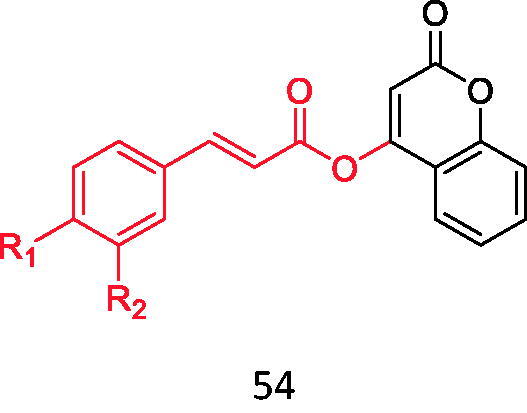
Compound **54**.

**Figure 10.**
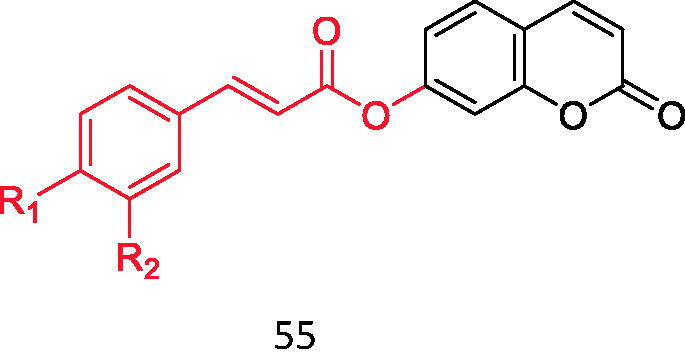
Compound **55**.

Cinnamic acid is a natural compound extracted from cinnamon oil. In recent years, the research of this kind of compound and its derivatives in the treatment of type 2 diabetes has become more and more extensive Based on this, Xu et al.^41^ combined substituted cinnamic acid with 4-hydroxycoumarin (Figure 9, Table 5) and 7-hydroxycoumarin (Figure 10, Table 6), and evaluated the inhibitory effects of these compounds on α-glucosidase. It was found that the inhibitory activity of these compounds on α-glucosidase was much higher than that of cinnamic acid and coumarin, and the inhibitory effect of substituted cinnamic acid combined with 4-hydroxycoumarin on α-glucosidase was better than that of 7-hydroxycoumarin. In addition, the introduction of electron-donating groups such as methyl can enhance its inhibitory activity. Molecular docking studies also confirmed that the synthesised derivatives can be effectively inserted into the active bag of α-glucosidase. Therefore, these compounds may be a promising α-glucosidase inhibitor.

**Figure 11.**
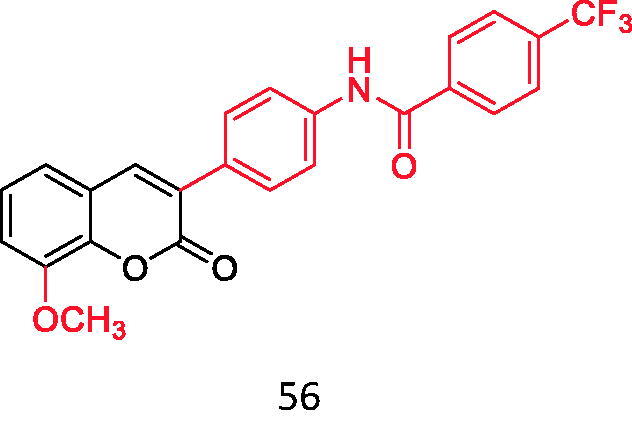
3-(4'-benzoyl amino-phenyl) coumarin derivatives.

**Figure 12.**
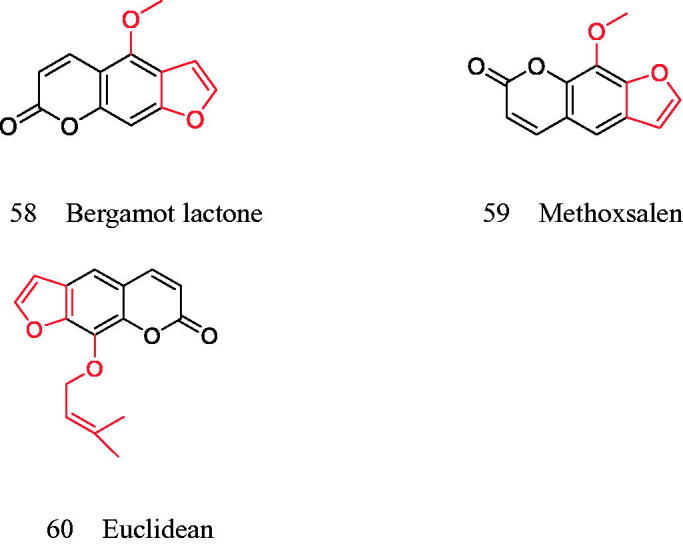
Coumarins for improving bone turnover and remodelling in diabetes.

**Figure 13.**
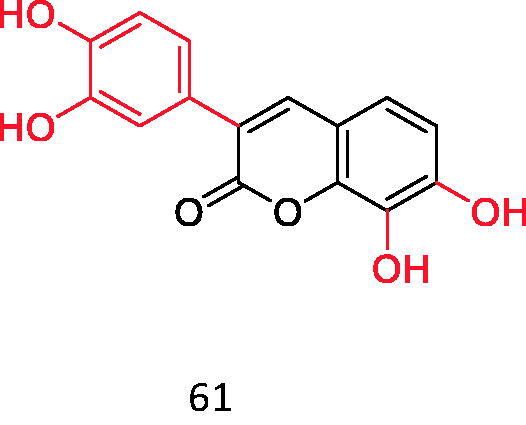
Coumarins with DPPH radical scavenging ability (o-catechol structure).

**Figure 14.**
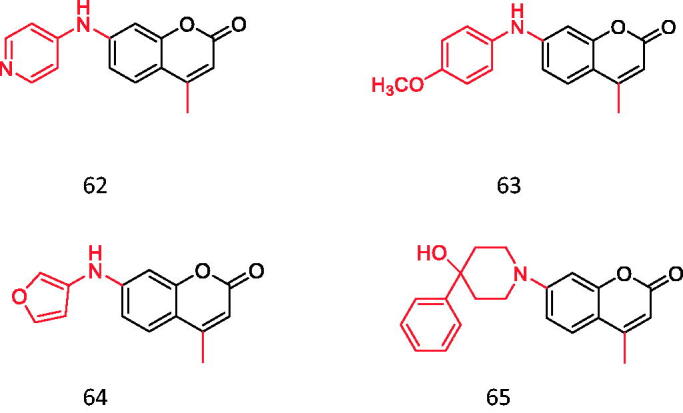
4-methyl-7-aminocoumarin and its derivatives.

**Figure 15.**
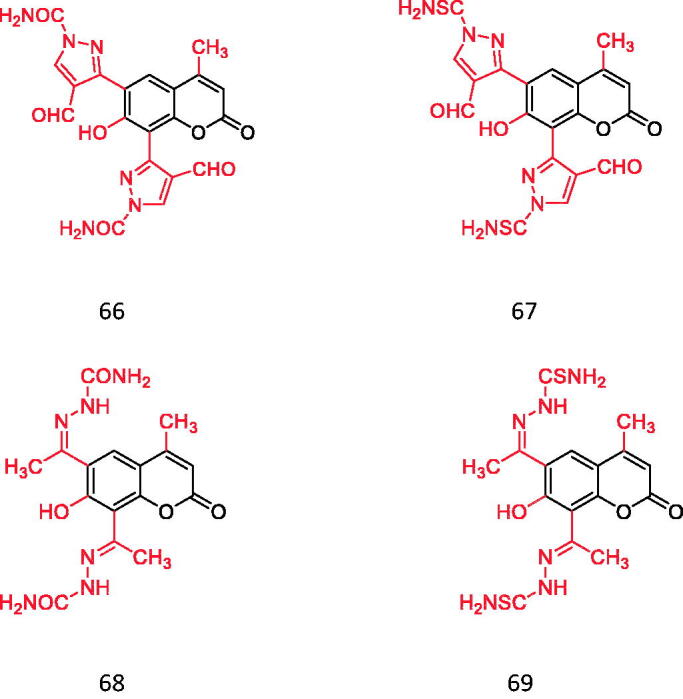
Pyrazole Cyclocoumarin (contains CONH_2_ and CSNH_2_).

**Figure 16.**
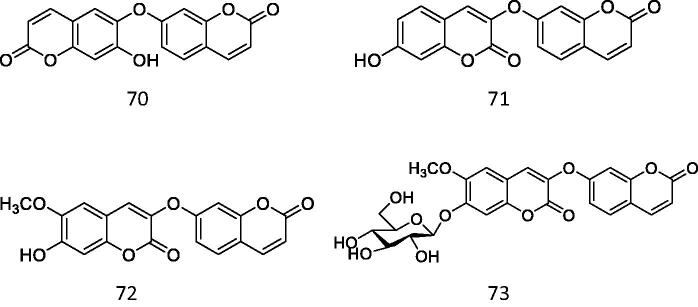
Coumarins that can activate Nrf2.

**Figure 17.**
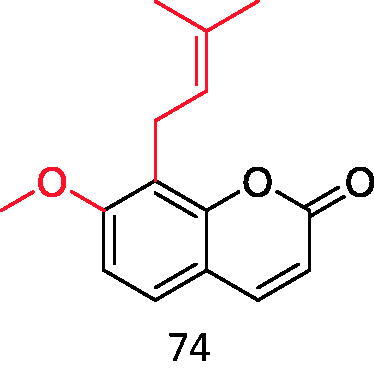
Osthole.

**Figure 18.**
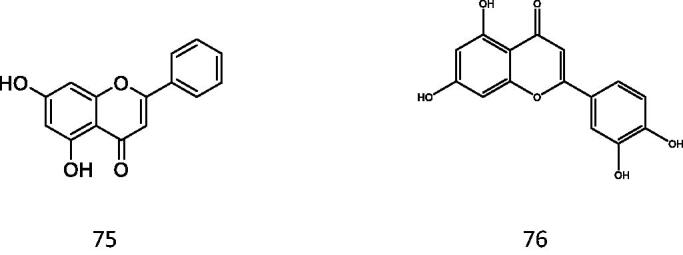
Chrysin and luteolin.

**Figure 19.**
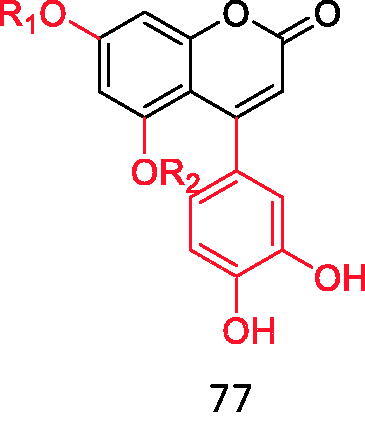
4-aryl coumarin.

**Figure 20.**
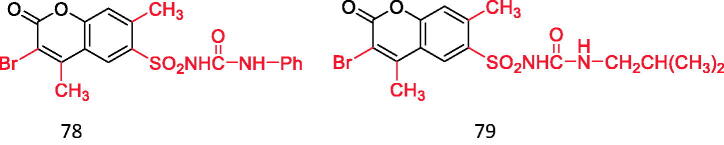
Coumarin binds to sulfonylurea structures.

**Figure 21.**
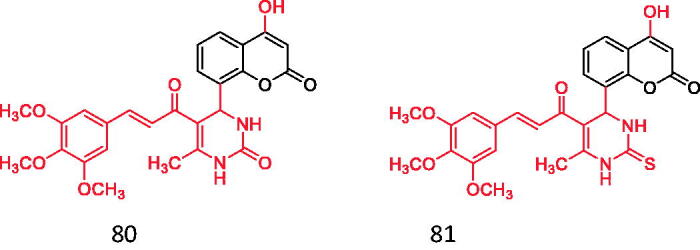
Coumarin chalcone hybrids.

**Table 5.** Binding of substituted cinnamic acid to 4-hydroxycoumarin.

Compound	*R*_1_	*R*_2_
**54-1**	H	H
**54-2**	CH_3_	H
**54-3**	OCH_3_	H
**54-4**	F	H
**54-5**	Cl	H
**54-6**	Br	H
**54-7**	CF_3_	H
**54-8**	OH	H
**54-9**	OH	OCH_3_

**Table 6.** Binding of substituted cinnamic acid to 7-hydroxycoumarin.

Compound	*R*_1_	*R*_2_
**55-1**	H	H
**55-2**	CH_3_	H
**55-3**	OCH_3_	H
**55-4**	F	H
**55-5**	Cl	H
**55-6**	Br	H
**55-7**	CF_3_	H
**55-8**	OH	H
**55-9**	OH	OCH_3_

#### Inhibition of advanced glycation end products (AGEs)

Advanced glycation end products are products of excessive sugar and protein combination, which is one of the hottest fields in the global medical field. Its formation is one of the key factors to induce diabetes and its complications^52^. Therefore, inhibiting the production of this substance has become one of the ideas for the treatment of diabetes and its complications. AGEs are the final products derived from the Maillard reaction, nonenzymatic glycation of free amino groups by sugars and aldehydes. Endogenous AGEs are mainly irreversible end products formed by non-enzymatic glycosylation of carbonyl and free amino groups in a hyperglycaemia environment, and isomerised by a series of reactions such as spontaneous rearrangement^53^^,^^54^. Hu^50^ studied 3–(4′-benzoyl amino-phenyl) coumarin derivatives and found that the inhibitory activity of these compounds on AGEs was generally higher than that of aminoguanidine hydrochloride, the positive control drug, especially the compounds shown in the figure below had the highest inhibitory activity, which was **58** times as much as that of its positive drug (Figure 11).

Through Wang's research on 3-aryl coumarin, it was found that most of the 3-aryl coumarins showed stronger AGEs inhibitory activity, and some of them were even stronger than the positive control drug aminoguanidine hydrochloride. Contrary to α-glucosidase inhibitory activity, 7-OH showed stronger AGEs inhibitory activity than 5,7-dihydroxy. Therefore, 3-aryl coumarins have great development potential in the treatment of diabetes (Table 7).

**Table 7.** Compound **57**, resveratrol and genistein have a comparative effect on a-glucosidase inhibitory activity and AGEs formation inhibitory activity.

Compound	Structure	α-glucosidase inhibition IC50 Value (μm)	AGEs formation inhibitory activity IC50 Value (μm)
Compound **57**	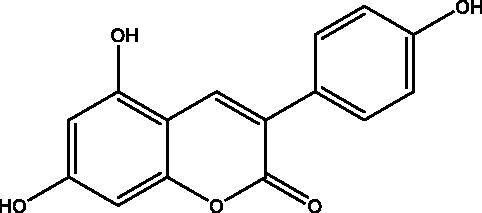	19.08 ± 0.26	3.12 ± 0,33
Resveratrol	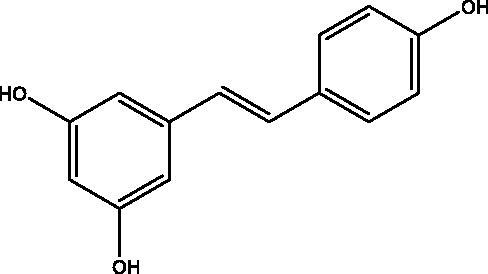	>1000	3.64 ± 0.92
Genistein	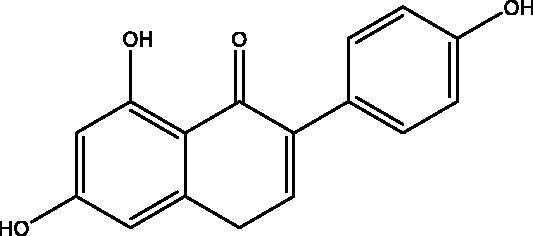	724.11 ± 39.74	11.04 ± 1.15

Patients with diabetes have an increased risk of osteoporotic fractures^55–57^. Therefore, diabetes-induced bone fragility has recently been recognised as a diabetic complication. Since the risk of fracture is not associated with a decrease in bone density, deterioration in bone quality may be a major cause of bone fragility^58^. In diabetic patients, persistent hyperglycaemia significantly increased AGE secretion and RAGE induction of MC3T3-E1 cells, and the interaction between AGE and RAGE destroyed osteoblast differentiation and bone formation, even during osteogenic differentiation. When the diabetic cells were treated with coumarin (≥10 μM), the production of AGE and RAGE decreased significantly. Thiazolidinediones, an antidiabetic drug^59^, can inhibit bone resorption of osteoclasts regardless of its lipid-forming effect, so these drugs have obvious shortcomings. Coumarins can improve bone turnover and bone remodelling in diabetic patients. Its derivatives bergamot lactone and methoxysarin can prevent diabetic osteoporosis by inhibiting osteoclast gene expression and bone absorption in diabetic bone tissue^60–62^; Imperatorin and bergamot lactone can enhance ALP activity, type I collagen synthesis, bone morphogenetic protein −2 expression and bone nodule formation in primary cultured osteoblasts and tibia tissue (Figure 12).

To sum up, AGE-RAGE signalling pathway plays a role in diabetic complications (including diabetic osteopathy)^63–66^, while coumarin inhibits this signalling pathway, which makes coumarin improve the molecules of bone turnover and bone remodelling.

#### Anti-oxidative stress

Free radicals are usually produced by a series of enzymatic or non-enzymatic oxidation reactions in organisms. Many free radicals are produced in the human body due to various metabolism. In the process of metabolising organic matter such as sugars, lipids and proteins, oxygen molecules (O_2_) generate water (H_2_O) mainly by four-step single-electron reduction under the action of the mitochondrial respiratory chain. In the complete reduction process of oxygen, it is necessary to first form the superoxide anion O_2_^−^, etc., and then combine with the hydrogen ion (H^+^) and finally generate water. However, it is not perfect, and some superoxide anions open small differences during the reduction reaction and are not completely reduced, but receive single or double electrons halfway through the respiratory chain and are partially reduced to generate superoxide and hydrogen peroxide (H_2_O_2_), That is, free radicals are produced. When overeating or hyperglycaemia occurs, a large number of energy substrates exceed the utilisation efficiency of mitochondria and many superoxides are produced. Redox homeostasis is crucial for maintaining physiological functions^67^. Oxidative stress refers to the imbalance between oxidative and antioxidant activities^68^. The formation of oxidative stress leads to the damage of islet β cells and insulin resistance and finally leads to the occurrence and development of diabetes and its complications. Therefore, antioxidant therapy is of great significance to delay the occurrence of diabetes and its complications.

DPPH is 1,1-diphenyl-2-trinitrophenylhydrazine, which is a stable free radical with a dark purple prismatic crystal. There are two main applications of DPPH: First, as a reaction monitoring substance in chemical reactions containing free radicals, DPPH is typically used to evaluate the antioxidant activity of antioxidant components *in vitro*; It can also be used as a standard material for the position and intensity of electron paramagnetic resonance signals. Wang studied the DPPH activity of 44 kinds of 3-aryl coumarins and found that the compounds with o-diphenol hydroxyl structure have stronger DPPH radical scavenging ability than its positive control vitamin C, especially the compounds shown below have the highest activity, and their radical scavenging activity is twice that of vitamin C (Figure 13).

Glucose can stimulate the intracellular production of ROS^69^. Physiologically, ROS promote insulin secretion, while excessive ROS can cause severe oxidative stress, downregulating the expression of the insulin protein, destroying pancreatic β cells and triggering insulin resistance^70^^,^^71^. It has been found that cardiovascular complications of diabetes are closely related to vascular calcification, and a large part of cellular calcification is due to the increase of total reactive oxygen species (ROS) content in cells. Muthipeedika et al.^72^ have studied the oxygen radical scavenging activity of a series of 4-methyl-7-aminocoumarin and its derivatives (Figure 14). The results show that among the 21 compounds studied, the existence of an electron-donating group or atom at the 7-position is an important feature of its enhanced antioxidant activity.

Hydroxycoumarin is an effective metal chelating agent, free radical scavenger and powerful chain-breaking antioxidant. Many coumarin derivatives have the unique ability to scavenge active oxygen, such as hydroxyl radical and superoxide to prevent free radical damage. The Mysore University of India, Georgia Institute of Technology and other institutions have attached pyrazole rings to hydrazine, hydrazine formamide and hydrazine methylthioamide coumarin to study their antioxidant activities. Its free radical scavenging ability was evaluated by DPPH and ROS scavenging experiments. They found that compounds **66** and **67** containing CONH_2_ and CSNH_2_ on the pyrazole ring had better antioxidant activity than ascorbic acid. The activity of compounds **68** and **69** in carbamates is lower than **66** and **67**, but it is better than ascorbic acid^73^ (Figure 15).

Diabetic nephropathy (DN) is a severe microvascular complication of diabetes mellitus^74^. Oxidative stress and fibrosis largely contribute to the progression of DN. According to the results of molecular docking by Huankai Yao et al., compound **73** can bind to Keap1 and significantly activate Nrf2. Cell-based assays have revealed compound **73** activated Nrf2 and attenuated oxidative stress and fibrosis induced by high glucose in mesangial cells^75^ (Figure 16).

Under high glucose, mesangial cells initiate self-limited proliferation at the early stage of DN, which is followed by glomerular hypertrophy and expansion. The viability of mesangial cells cultured in a normal or high glucose medium was evaluated by CCK-8 analysis. There was no significant inhibitory effect of compound **73** on mesangial cells under normal conditions. However, high glucose-stimulated the proliferation of mesangial cells and led to an increase in their vitality. For compounds **70–72**, at 10 μM they began to inhibit the proliferation stimulated by high glucose significantly though they didn’t show toxicity to normal mesangial cells even at 100 μM. However, at 5 and 10 microns, compound **73** significantly reduced the increased viability. Western blot analysis and following densitometric analysis also revealed the Nrf2 was activated by compound **73** in mesangial cells under high glucose. Compound **73** as an Nrf2 activator attenuated oxidative stress and fibrosis induced by high glucose in mesangial cells through disrupting the interaction between Keap1 and Nrf2. This investigation can provide evidence for further investigations on compound **73** in vivo and the discovery of new drugs targeting DN.

#### Up-regulated expression of P2X3 after treatment

Diabetic neuralgia (DNP) is one of the most common complications of diabetes^76^. DNP is related to the enhancement of peripheral sensory nerve excitability, involving various ion channels, receptor expression and up-regulation of function. P2X3 receptor is a member of P2X family of purine receptors and participates in many neuropathological pain processes including DNP. P2X3 receptor is a non-selective ligand-gated cation channel, which is mainly distributed in some sympathetic neurons, sensory neurons and nucleus tractus solitarius, mainly in small and medium ganglion cells. P2X3 receptor increases pain when its expression is up-regulated or its activity is enhanced and decreases pain when its expression is down or desensitised, indicating that P2X3 receptor is an important receptor for progressive pain^77^.

Wu et al. studied that osthol inhibited the expression of P2X3 receptor in stellate sympathetic ganglion (SG) of diabetic rats (Figure 17). They studied by immunohistochemistry and western blot and found that the integrated optical density (IOD) in the diabetic group was significantly higher than that in the control group. Compared with a diabetic group, the expression of P2X3 in the osthol group decreased significantly, which indicated that osthol treatment could resist the up-regulation of P2X3 expression in SG of diabetic rats^78^.

#### Activate PPAR-γ

Among the three subtypes of PPARs, peroxisome proliferator-activated receptor γ (PPAR-γ) is the most reported, which is the key regulator of glucose metabolism^79–82^. Chrysin and luteolin are two flavonoids with PPAR-γ stimulatory activity that protect against vascular complications associated with insulin resistance (IR) (Figure 18). Meanwhile, chrysin and luteolin significantly inhibited NO and ROS elevation in IR aortas. In conclusion, chrysin and luteolin alleviate IR-related vascular complications mainly through the PPAR-γ-dependent pathway. Coumarins are expected to improve cardiovascular problems of diabetic patients in PPAR-γ agonistic activity because of their high structural similarity with flavonoids^83^.

Positive effects of preparations of the bark of the Central American plant Hintonia latiflora (family Rubiaceae) on blood glucose reduction and therefore the maintenance of physiologically normal blood glucose values have been reported^84–86^. Jose et al. conducted mouse experiments on 4-aryl coumarin glycosides extracted from Hintonia latiflora, and found that the blood glucose concentration of diabetic mice decreased obviously and the insulin level showed an upward trend after injection of this compound. This experiment shows that 4-aryl coumarin glycosides have obvious hypoglycaemic activity (Figure 19). Its structural formula is as follows:

The 7th position can be hydroxyl or methoxy, and R_2_ can be glucopyranoside, galactopyranoside, etc.

Tu et al.^87^ found that the introduction of sulfonylurea structure into coumarin mother nucleus showed good hypoglycaemic activity, besides, it also showed good antibacterial activity, which is expected to become a new hypoglycaemic drug different from sulfonylureas. Among the 12 selected compounds, the hypoglycaemic activity of the following two compounds is equivalent to that of positive control drugs (compounds **78** and **79**) (Figure 20).

#### Insulin receptor

Type II diabetes mellitus is one type of DM found in more than 90% of cases of DM and could be attributed to obesity, overweight and lack of physical activity, marked by pancreatic insulin release, when the body has not been trained to utilise insulin developed for glucose transfer, and the emergence of insulin resistance contributes to an increase of blood glucose or hyperglycaemia.^88^ Therefore, the insulin receptor is a potential target for screening the anti-diabetic ligand activity of insulin receptor activator, and it is a tyrosine kinase transmembrane receptor, which effectively participates in the regulation of glucose homeostasis through phosphorylation of insulin binding^89–92^. A total of 54 coumarin chalcone hybrids were synthesised by the famous Biginelli synthesis, Pechmann condensation, acetylation and Claisen-Schmidt reaction. Compared with diabetic rats treated with metformin (100 mg/kg b.d), further treatment with 80 and 81 at 30 mg/kg b.d. showed that MDA in pancreas and liver tissue of diabetic rats decreased significantly and moderately, while SOD and GSH rates increased^89^ (Figure 21).

## Summary and prospect

The research on the therapeutic effect of coumarins in diabetes is deepening. This review summarises the hypoglycaemic activity of coumarins and their derivatives, which is conducive to the design of coumarins and their derivatives with more significant efficacy by medicinal chemists.

Among the various coumarin compounds mentioned above, 3-arylcoumarin derivatives hold promise as candidate molecules for antidiabetic drugs and further studies. Among them, compounds with hydroxyl structures at position 7 had α-glucosidase inhibitory activity; compounds with hydroxyl groups in 5,7-dihydroxyl and 4'-OH structures also had significant α-glucosidase inhibitory activity; and the above 3-arylcoumarins had AGEs inhibitory activity. The structure containing catechol hydroxyl group can effectively clear the DPPH radical; 4-methyl-7-aminocoumarins and dicoumarins also have oxygen radical scavenging activity; osthole slows down the occurrence and development of DNP, a diabetic complication, by resisting the expression of P2X3 in diabetic rats; coumarin chalcone hybrids have a significant effect on insulin resistance.

In conclusion, by summarising the hypoglycaemic activity of different structures of coumarins as well as the synthesis methods of some of them, it provides a reference for further study of the hypoglycaemic effect of coumarin compounds and opens up new horizons.

## Ethics statement

We further confirm that any aspect of the work covered in this manuscript that has involved human patients has been conducted with the ethical approval of all relevant bodies and that such approvals are acknowledged within the manuscript.
